# Combined Assessment of the Tumor–Stroma Ratio and Tumor Immune Cell Infiltrate for Immune Checkpoint Inhibitor Therapy Response Prediction in Colon Cancer

**DOI:** 10.3390/cells10112935

**Published:** 2021-10-28

**Authors:** Cor J. Ravensbergen, Meaghan Polack, Jessica Roelands, Stijn Crobach, Hein Putter, Hans Gelderblom, Rob A. E. M. Tollenaar, Wilma E. Mesker

**Affiliations:** 1 Department of Surgery, Leiden University Medical Center, Albinusdreef 2, 2300RC Leiden, The Netherlands; c.j.ravensbergen@lumc.nl (C.J.R.); m.polack@lumc.nl (M.P.); R.A.E.M.Tollenaar@lumc.nl (R.A.E.M.T.); 2 Department of Pathology, Leiden University Medical Center, Albinusdreef 2, 2300RC Leiden, The Netherlands; j.p.roelands@lumc.nl (J.R.); a.s.l.p.crobach@lumc.nl (S.C.); 3 Department of Medical Statistics, Leiden University Medical Center, Albinusdreef 2, 2300RC Leiden, The Netherlands; h.putter@lumc.nl; 4 Department of Medical Oncology, Leiden University Medical Center, Albinusdreef 2, 2300RC Leiden, The Netherlands; A.J.Gelderblom@lumc.nl

**Keywords:** tumor–stroma ratio, colon cancer, tumor-infiltrating immune cells, immunotherapy, tumor microenvironment, checkpoint inhibitor

## Abstract

The best current biomarker strategies for predicting response to immune checkpoint inhibitor (ICI) therapy fail to account for interpatient variability in response rates. The histologic tumor–stroma ratio (TSR) quantifies intratumoral stromal content and was recently found to be predictive of response to neoadjuvant therapy in multiple cancer types. In the current work, we predicted the likelihood of ICI therapy responsivity of 335 therapy-naive colon adenocarcinoma tumors from The Cancer Genome Atlas, using bioinformatics approaches. The TSR was scored on diagnostic tissue slides, and tumor-infiltrating immune cells (TIICs) were inferred from transcriptomic data. Tumors with high stromal content demonstrated increased T regulatory cell infiltration (*p* = 0.014) but failed to predict ICI therapy response. Consequently, we devised a hybrid tumor microenvironment classification of four stromal categories, based on histological stromal content and transcriptomic-deconvoluted immune cell infiltration, which was associated with previously established transcriptomic and genomic biomarkers for ICI therapy response. By integrating these biomarkers, stroma-low/immune-high tumors were predicted to be most responsive to ICI therapy. The framework described here provides evidence for expansion of current histological TIIC quantification to include the TSR as a novel, easy-to-use biomarker for the prediction of ICI therapy response.

## 1. Introduction

The tumor microenvironment (TME), or tumor stroma, refers to the local environment in which malignant cells are embedded, and comprises a multitude of (sub)cellular components [1]. The dynamic interactions that occur between the TME and malignant cells promote tumorigenesis and are essential in cancer progression [2]. The tumor–stroma ratio (TSR), previously discovered by our research group, is a prognostic tool that stratifies patients into high-risk and low-risk categories based on the histologic quantification of stromal content within the primary tumor (PT) [3]. Tumors with a high stromal content were previously shown to have a poor patient outcomes in a variety of epithelial malignancies [4,5,6,7,8,9,10,11]. In addition, the TSR was found to be a predictor of pathologic response to neoadjuvant therapy in breast and esophageal cancer [12,13,14].

Molecular modulation of the tumor stroma, as a key driver of cancer progression and dissemination, has gained scientific interest over the past few years [15,16]. Considering the molecular background of the TSR, our understanding of the molecular drivers behind its prognostic value is gradually advancing. We recently identified a prognostic gene expression ratio of stromal versus epithelial genes that correlates to the TSR [17]. Moreover, cell membrane and gene expression markers associated with cancer-associated fibroblasts (CAFs) were found to be increased in stroma-high tumors, compared with stroma-low tumors [18]. Given the key role of CAFs in extracellular matrix (ECM) deposition and remodeling and their association with poor prognosis, CAF enrichment in the TME is likely to be a driver of high stromal content [19].

Besides mesenchymal cells, the immune system is considered to be a crucial component of the tumor stroma, where tumor-infiltrating immune cells (TIICs) host a variety of pro-tumorigenic and anti-tumorigenic roles [20]. Considering the interplay between the immune system and the TSR, we recently observed a synergistic effect of high stromal content and intratumoral HLA class I expression on patient survival, in which patients with a stroma-high tumor and concurrent low HLA class I expression demonstrated poorer survival rates [21]. Moreover, stroma-high tumors were associated with increased infiltration of selected macrophage and T cell subsets in breast cancer [22]. Nevertheless, a comprehensive characterization of TIIC composition and its clinical relevance to intratumoral stromal content is currently lacking.

Recently, advances in immune-system-derived treatment modalities, such as immune checkpoint inhibitor (ICI) therapy, have resulted in a therapeutic paradigm shift in clinical oncology [23]. Despite the clinical success of ICI therapy, the considerable interpatient variability in objective response rates remains a major challenge, with an estimated percentage of responders across eligible primary tumor types of approximately 13% in U.S. patients in 2018 [24]. To maximize therapeutic benefit and avoid unnecessary toxicity, the establishment of predictive biomarkers to guide the treatment decision-making process is warranted. Over the past few years, multiple biomarker strategies have been proposed as predictors of ICI therapy response; amongst others, tumor-infiltrating lymphocyte density, microsatellite instability (MSI), immunohistochemistry-based PD-L1 expression, and tumor mutational burden (TMB) [25]. However, a cumulative number of reports demonstrate ambiguous results in the application of these biomarkers [26,27].

Given its recent discovery as a predictor of response to (neo)adjuvant therapy and the key role of the ECM in TIIC composition, we hypothesize that the TSR may have additive clinical value in predicting response to ICI therapy. In the current work, we set out to explore the TIIC composition in stroma-high and stroma-low colon carcinoma tumors using bioinformatics approaches. We subsequently define a stromal classification, based on intratumoral stromal content and TIIC composition, which has been associated with previously established transcriptomic and genomic biomarkers for ICI therapy response.

## 2. Materials and Methods

### 2.1. Data Acquisition

We analyzed gene expression profiles obtained from The Cancer Genome Atlas (TCGA). Illumina HiSeq Level 3 mRNA bulk sequencing data and clinical metadata from the TCGA colon adenocarcinoma (COAD) project were obtained from the Genomic Data Commons (GDC; https://gdc.cancer.gov, accessed on 1 June 2021) by R/Bioconductor package TCGAbiolinks (version 2.18.0) [28]. Samples were included based on colon adenocarcinoma histological subtype and complete microsatellite status data (*n* = 366). Patient identifiers of the included cohort are available in Supplementary Data S1. Absolute gene expression data were gene length normalized, adjusted for within-lane and between-lane effects using Python package HTSeq, and expressed as fragments per kilobase million mapped reads (FPKM) [29].

For the validation cohort, we analyzed gene expression profiles (*n* = 106) from the Clinical Proteomic Tumor Analysis Consortium (CPTAC) prospective proteogenomic analysis of colon adenocarcinoma [30]. Level 3 mRNA bulk sequencing data and clinical metadata from the CPTAC cohort were obtained from the LinkedOmics repository (http://linkedomics.org/login.php, accessed on 1 June 2021). Absolute gene expression data were gene length normalized and expressed as transcripts per million mapped reads (TPM). Patient identifiers of the included cohort are available in Supplementary Data S1.

### 2.2. CIBERSORTx Digital Cytometry

The CIBERSORTx web portal (https://cibersortx.stanford.edu/, accessed on 1 July 2021) was utilized to run the validated 22-phenotype leukocyte signature (LM22) in absolute mode with 100 permutations and B-mode batch correction [31]. The LM22 signature infers immune cell populations based on the expression of 547 immune cell-expressed genes; a list of the 22 leukocyte phenotypes can be found in Supplementary Table S1. A total of 6 (1.1%) of the 547 genes of the LM22 signature matrix were missing from the input mRNA sequencing data. The CIBERSORTx absolute mode scales relative cellular fractions into an absolute score that reflects the absolute proportion of each cell type in a tumor, which can subsequently be compared amongst cell types [32]. Gene-length normalized FPKM gene expression data were used as input data. Samples with accurate CIBERSORTx deconvolution (*p* < 0.05) were considered to be eligible for further immunophenotyping analysis. For assessment of relative intra-tumoral TIIC heterogeneity, absolute scores were normalized to 1.

### 2.3. Tumor–Stroma Ratio

The TSR was scored on digital diagnostic hematoxylin and eosin (H&E)-stained slides of primary tumors from the TCGA COAD project, retrieved from the GDC portal. The TSR was scored using Aperio Imagescope (version 12.4.3) digital slide viewer software. The area with the highest amount of stroma was selected, according to the previously published protocol for colon cancer; a detailed description of the methodology and scoring eligibility criteria can be found in this protocol [33]. The percentage of stroma was scored in increments per ten percent (i.e., 10%, 20%, etc.) and the tumor was subsequently categorized as a stroma-high (>50%) or stroma-low (≤50%) tumor. Observers (CR and MP) were trained with the TSR E-learning module constructed for the Uniform Noting for International Application of the Tumor-Stroma Ratio as an Easy Diagnostic Tool (UNITED) study [34,35]. In 33% percent of the slides, blinded visual scoring was performed by a second observer; subsequently, the interobserver agreement was assessed by Cohen’s kappa coefficient. When consensus could not be reached, the assessment of a third observer (S.C., board-certified pathologist) was decisive.

### 2.4. Definition of Stromal Categories

We defined 4 stromal categories based on histological stromal content and molecular-derived immune cell infiltration data. First, the TSR was used to categorize patients in stroma-low and stroma-high groups as described in detail above. Next, the 2 groups were further categorized based on total immune cell infiltration, as computed by the CIBERSORTx absolute mode, by using the median of the total cohort as a cut-off value. The resulting stromal categories were a combination of intra-tumoral stromal content and total immune cell infiltration. Due to a lack of tissue slides in the validation cohort, we stratified intra-tumoral stromal content based on the stromal score of the Estimation of STromal and Immune cells in MAlignant Tumours using Expression data (ESTIMATE) computational method for tumor purity [36], described elsewhere in the methods section. To secure maximum comparability in the validation cohort, we applied the same stroma-high and stroma-low distribution as scored by the TSR in the discovery cohort. ESTIMATE’s immune score was used as a surrogate to CIBERSORTx immune quantification and the validation cohort was stratified into immune-high or immune-low groups by the median immune score of the total cohort.

### 2.5. Microsatellite Instability, Tumor Mutational Burden, and Single Nucleotide Variants

Microsatellite status data for the TCGA COAD cohort were obtained through the GDC portal (https://gdc.cancer.gov, accessed on 1 June 2021). Data for the CPTAC validation cohort were obtained at the LinkedOmics online repository (http://linkedomics.org/login.php, accessed on 1 June 2021). A detailed description of MSI testing methodology can be found in the methods section of the original publications [30,37]. Precomputed tumor mutational load data were acquired from the Supplementary Materials of the TCGA Immune Landscape of Cancer publication [38]. Mutational load was defined as the sum of silent and non-silent mutations per megabase (Mb). Using the recently FDA-approved cut-off of 10 mutations per megabase (mut/Mb), the tumors were categorized into a TMB-low (TMB-L, ≤10 mut/Mb) and a TMB-high (TMB-L, >10 mut/Mb) group for further analysis [39]. As a genomic classifier in the CPTAC validation cohort, we analyzed synonymous and non-synonymous single nucleotide variants (SNV).

### 2.6. MIRACLE and TIDE Prediction Scores

The Mediators of Immune Response Against Cancer in soLid microEnvironments (MIRACLE) score is a novel computational approach to predict response to ICI therapy without the need for dataset-specific normalization [40]. A detailed description of the MIRACLE score and its methodology can be found in the respective publication. In short, the MIRACLE score integrates stimulatory and suppressive immunological signals, derived from transcriptomic data, into a balance score that captures the local immune landscape present in the tumor. MIRACLE scores were computed using the web application (available at: https://miracle.shinyapps.io/miracle_shinyapp/, accessed on 1 June 2021) with gene expression profiles as input.

In addition to MIRACLE, the Tumor Immune Dysfunction and Exclusion (TIDE) algorithm is a computational method to predict response to ICI therapy by modeling two primary mechanisms of tumor immune evasion—namely the induction of T cell dysfunction, depicted by a dysfunction signature, and the prevention of tumor infiltration, depicted by an exclusion signature [41]. Precomputed TIDE signature scores for the TCGA data were obtained through the TIDE web portal (http://tide.dfci.harvard.edu/, accessed on 1 June 2021). The TIDE prediction score was subsequently generated as the standard-deviation-normalized dysfunction score for the tumor samples with high cytotoxic-lymphocyte (CTL) infiltration, and as the standard-deviation-normalized exclusion score for the tumor samples with low CTL infiltration, as per recommendations given by the original authors [41]. Tumors were categorized as CTL-high if the average expression of CTL markers (CD8A, CD8B, GZMA, GZMB, and PRF1) per sample was greater than the average expression of these markers in the total cohort. The remaining tumors were categorized as CTL-low tumors. For the validation cohort, de novo TIDE prediction scores were computed using the TIDE web portal. The gene expression matrix was normalized by the average expression value per gene. All computed scores in this study can be found in Supplementary Data S1.

### 2.7. Gene Set Enrichment Analysis

Single sample gene set enrichment analysis (ssGSEA) was performed on the normalized gene expression data to define gene enrichment of specific stromal and immune pathways [42]. The TGF-β and CXCR4 signaling pathway gene sets used in this study were obtained from the Molecular Signatures Database (MSigDB) [43]. The checkpoint genes included in the checkpoint gene set were selected based on a literature search. All gene sets used in this study are available in Supplementary Data S1.

### 2.8. Statistical Analysis

The R programming language (version 4.0.5; https://www.r-project.org/, accessed on 1 June 2021) was used for statistical analysis and data visualization (packages EDASeq, tidyverse, viridis, corrplot, ggExtra, GSVA, factoextra, and igraph). Variable distribution was evaluated with the Shapiro–Wilk test. For comparison analysis, Fisher’s exact test or the Chi-squared test were used for categorical variables and the Mann–Whitney U test was used for continuous variables, following the assessment of variable distribution. Likewise, parametric (Pearson’s r) or non-parametric (Spearman’s rho) correlation coefficients were computed, depending on the variable distribution. For comparative analysis, gene expression values were log base 2 transformed. The expression of the checkpoint genes was summarized by computing the geometric mean expression per tumor sample. A two-tailed *p*-value of ≤0.05 was considered statistically significant.

## 3. Results

### 3.1. Sample Characteristics and TIIC Composition

We selected 366 gene expression profiles from the TCGA COAD project for digital cytometric analysis with CIBERSORTx. Patients were selected based on adenocarcinoma histological subtype and complete microsatellite status data. All patients were therapy-naive upon data acquisition. Additional patient characteristics can be found in Supplementary Table S2. Using CIBERSORTx, immune cell composition could be accurately inferred (*p* < 0.05) in 359 samples, which were subsequently included for further analysis in this study. In the remaining seven samples, the imputed cell fractions did not differ from cell fractions obtained by random chance (*p* > 0.05) and were therefore excluded from further analysis.

**Figure 1:**
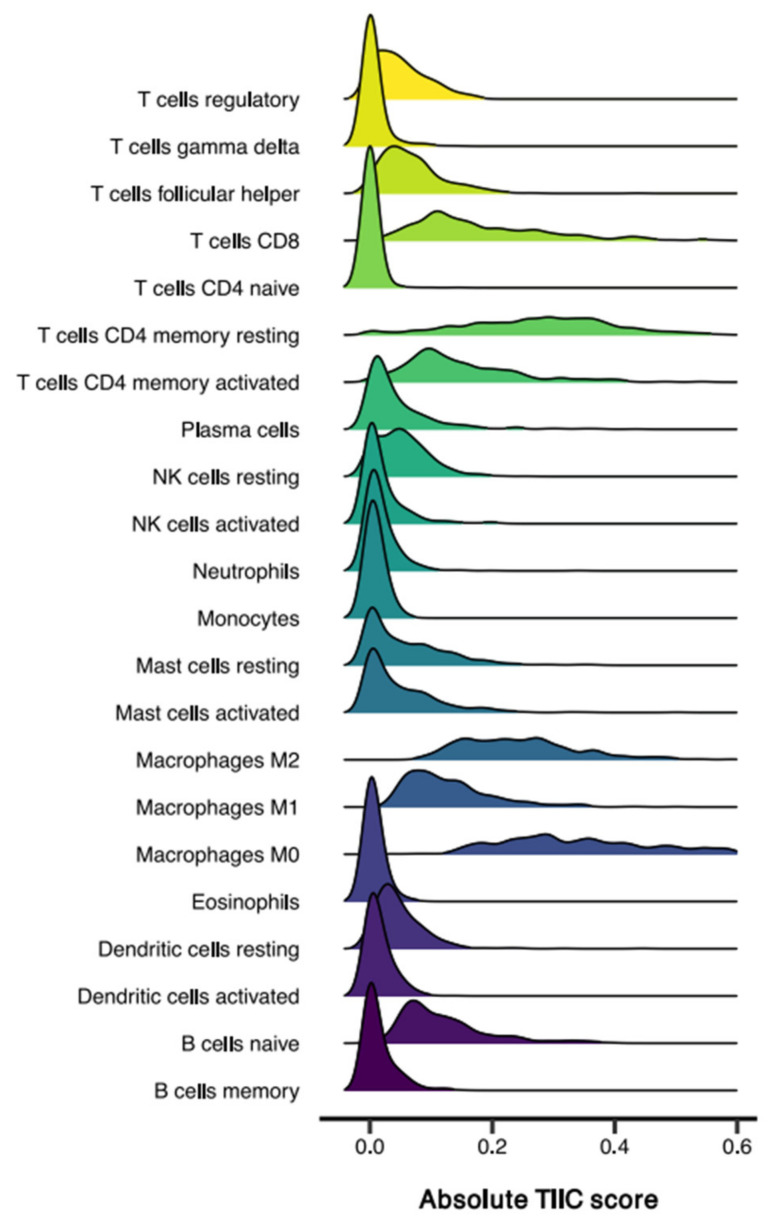
Distribution of tumor-infiltrating immune cell (TIIC) subsets in 359 colon adenocarcinoma tumors. Ridgeline plot of absolute infiltration of the 22 TIIC subsets, as defined by CIBERSORTx.

We first analyzed the relative composition of TIIC subsets in the total cohort of colon adenocarcinoma patients. Interestingly, mean relative percentages for the presence of TIICs of lymphoid and myeloid origin were comparable (50.7% and 49.3%, respectively). Likewise, TIIC proportions, classified on functional annotation of the immune system, were similar (adaptive 53.0% vs. innate 47.0%). Despite evident intertumoral heterogeneity in immune cell infiltration, the five most abundant cell subsets with the highest absolute TIIC score were M0 macrophages (median absolute TIIC score 0.357), resting CD4 T memory cells (0.285), M2 macrophages (0.239), CD8 T cells (0.158), and activated CD4 T memory cells (0.125; Figure 1).

### 3.2. Tumor Microsatellite Status Relates to Immune Cell Infiltration but Is Not Associated with Stromal Content

**Figure 2:**
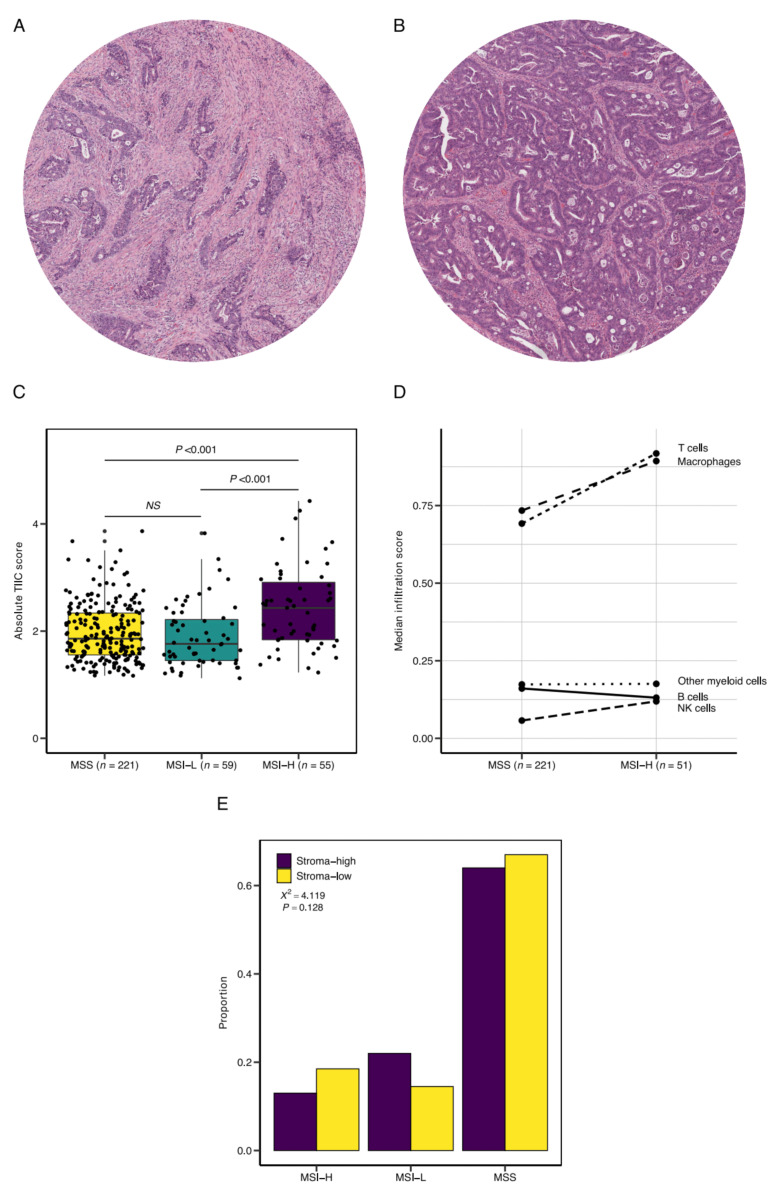
Tumor-infiltrating immune cells (TIICs) and microsatellite status. Representative illustrations of (**A**) stroma-high and (**B**) stroma-low tumors, as scored by the tumor–stroma ratio (TSR). (**C**) Total TIIC infiltration in microsatellite stable (MSS), low microsatellite instability (MSI-L), and high microsatellite instability (MSI-H) tumors. (**D**) Specification of 5 large TIIC subsets in MSS versus MSI-H tumors. (**E**) Proportion of stroma-high and stroma-low tumors in the microsatellite status subgroups. NS, non-significant; X2, chi-squared test.

Given the clinical association of DNA mismatch repair deficiency and response to ICI therapy, we then aimed to assess the TIIC composition and microsatellite status in stroma-high and stroma-low tumors. A total of 335 tumors were eligible for TSR scoring and were included for further analysis, as slides from nine (2.5%) tumors were not available (Supplementary Figure S1). The Cohen’s kappa coefficient for interobserver variability was 0.85, indicating near-perfect agreement. A third review by an independent observer was necessary to reach a complete agreement in 12 (3.5%) slides. Illustrative images of stroma-high and stroma-low tumors can be found in Figure 2A,B. Baseline characteristics of the stroma-low (*n* = 200, 59.7%) and stroma-high (*n* = 135, 40.3%) tumors can be found in Supplementary Table S3.

We observed a significant increase in absolute infiltration of total TIICs; i.e., the 22 measured immune cell phenotypes combined, in MSI-high (MSI-H) in comparison to MSI-low (MSI-L) and microsatellite stable (MSS) tumors (median 2.43 vs. 1.76 vs. 1.86, *p* < 0.001; Figure 2C). Upon closer inspection, the expansion of the TIICs in MSI-H tumors was largely attributed to the enrichment of T cell (median 0.92 vs. 0.69, *p* < 0.001) and macrophage (median 0.89 vs. 0.73, *p* = 0.006) populations, which demonstrated the largest increase in median infiltration score in comparison to MSS tumors (Figure 2D). Microsatellite status was not associated with stromal content (Figure 2E).

### 3.3. Stroma-High Tumors Demonstrate Increased Infiltration of T Regulatory Cells but Are Not Associated with Increased Expression of T Cell Exhaustion Markers

**Figure 3:**
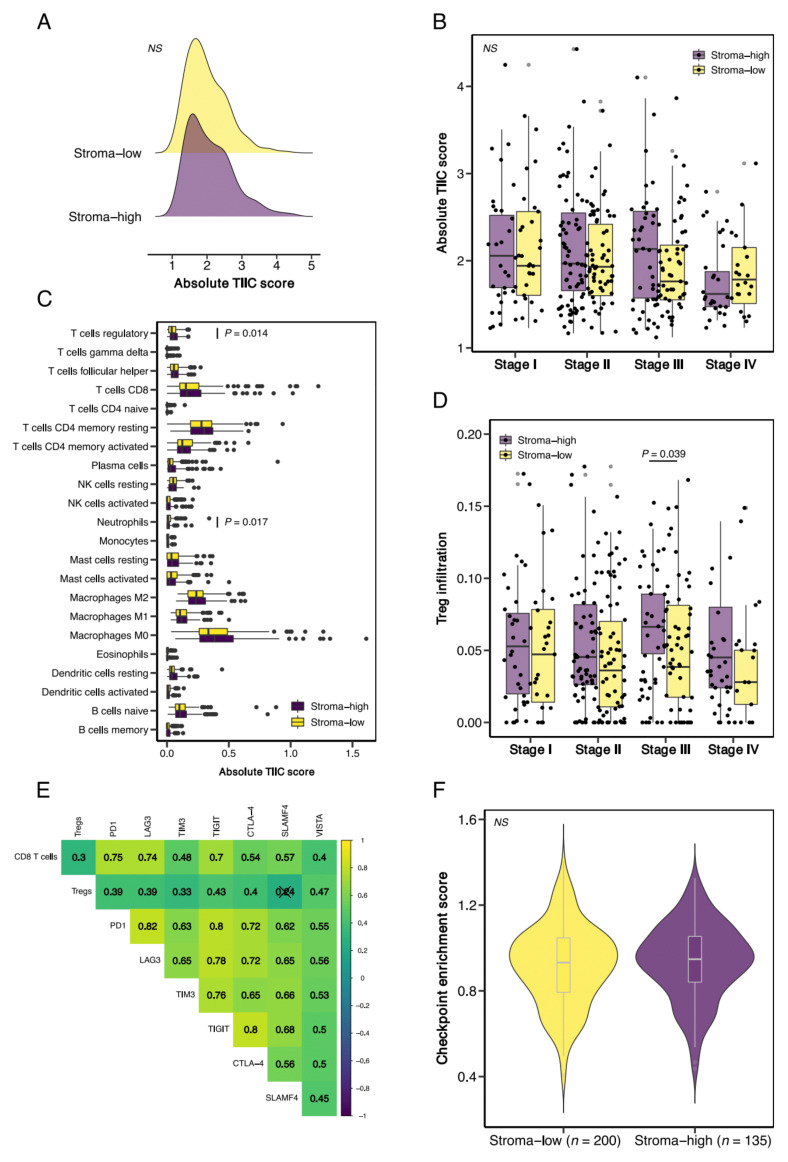
The tumor–stroma ratio (TSR), T regulatory cells (Tregs), and T cell exhaustion markers. (**A**) Total TIIC infiltration in stroma-high and stroma-low tumors, as scored by the TSR. (**B**) Total TIIC infiltration, stratified by tumor stage. (**C**) Specification of the infiltration of 22 immune cell subsets in stroma-high and stroma-low tumors. (**D**) Treg infiltration stratified by tumor stage. (**E**) Correlation matrix of CD8 T cells, Tregs, and canonical T cell exhaustion markers. (**F**) Gene set enrichment analysis of 7 checkpoint genes (i.e., PD1, LAG3, TIM3, TIGIT, CTLA-4, SLAMF4, and VISTA) in stroma-high and stroma-low tumors. NS, non-significant.

We then studied TIIC composition in stroma-low and stroma-high tumors and detected a nearly identical distribution of total TIIC scores (Figure 3A). In addition, we observed no significant differences in total TIICs in stroma-low versus stroma-high tumors stratified by tumor stage (Figure 3B). Subsequent analysis of the infiltration of the 22 distinct immune cell subsets demonstrated increased infiltration of T regulatory (Treg) cells (*p* = 0.014) and decreased infiltration of neutrophils (*p* = 0.017) in the stroma-high versus the stroma-low tumors (Figure 3C). When stratified by tumor stage, Treg infiltration was only significantly different in stage III (*p* = 0.039); however, stroma-high tumors in all stages demonstrated a trend towards increased Treg infiltration (Figure 3D).

Since Tregs are believed to host a key role in the suppression of antitumor immunity, we then tested whether increased Treg infiltration was associated with the expression of canonical T cell exhaustion markers PD-1, LAG3, TIM3, TIGIT, CTLA-4, SLAMF4, and VISTA [44]. High expression of these inhibitory markers can be identified on T cells with a poor effector function [45]. We found significant (*p* < 0.05) positive correlations for Treg infiltration and expression of all exhaustion markers, except SLAMF4, indicative of dysfunctional T cell effector function and an immune-suppressive TME. In addition, CD8 T cell infiltration was associated with both Treg infiltration and checkpoint expression (Figure 3E). These findings support previous reports on checkpoint expression, suggesting that increases in immune cell infiltration can be accompanied by paradoxical activation of immune-suppressive pathways, such as checkpoint expression [45,46]. Despite the increased infiltration of Tregs in stroma-high tumors, we did not observe significant differences in checkpoint gene enrichment scores between stroma-high and stroma-low tumors (Figure 3F). The same trend was observed when we analyzed the expression of the checkpoint genes separately (Supplementary Figure S2).

### 3.4. Defining Stromal Categories Based on Stromal Content and Immune Cell Infiltrate That Are Predictive of Response to ICI Therapy

**Figure 4:**
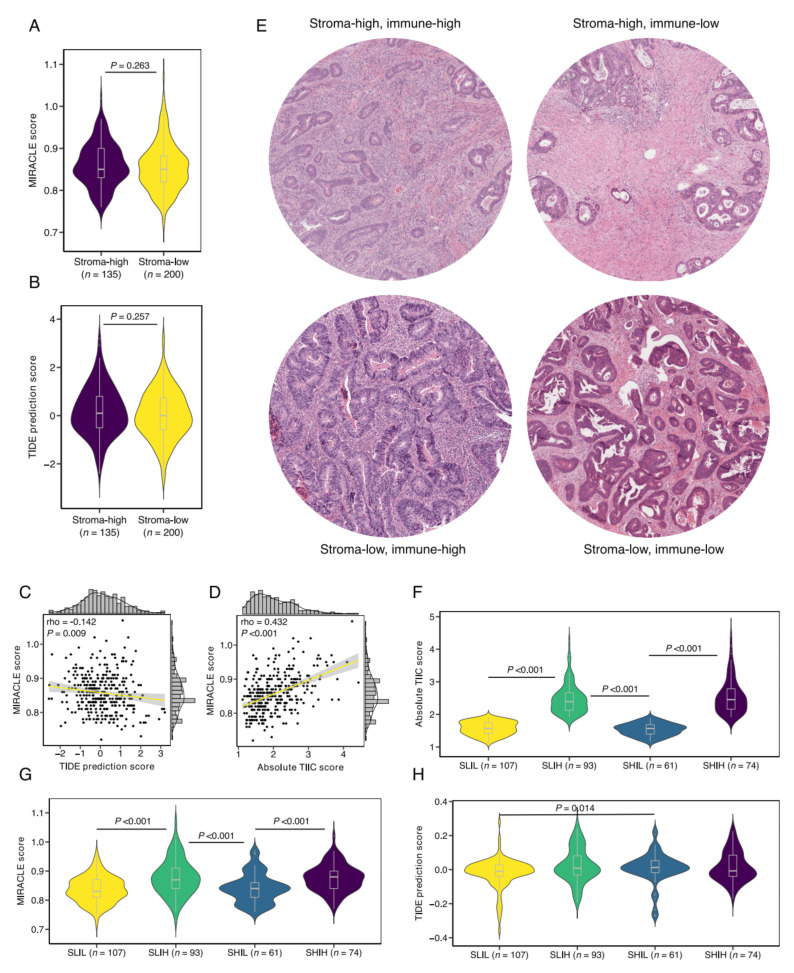
Defining stromal categories based on stromal content and immune cell infiltration. (**A**) MIRACLE scores in stroma-high and stroma-low tumors. (**B**) TIDE prediction scores in stroma-high and stroma-low tumors. (**C**) Correlation plot of MIRACLE and TIDE prediction scores. (**D**) Correlation plot of MIRACLE scores and total tumor-infiltrating immune cells (TIICs). (**E**) Representative illustrations of newly defined stromal categories based on stromal content and total TIICs. (**F**) The stromal categories and total TIICs. (**G**) The stromal categories and MIRACLE scores. (**H**) The stromal categories and TIDE prediction scores. Rho, Spearman’s rho; SLIL, stroma-low/immune-low; SLIH, stroma-low/immune-high; SHIL, stroma-high/immune-low; SHIH, stroma-high/immune-high.

We aimed to assess the role of the TSR in predicting response to ICI therapy. The recently established MIRACLE score is a computational approach for predicting response to ICI therapy, where a high MIRACLE score predicts favorable therapy response [40]. MIRACLE scores were computed per tumor sample and compared between stroma-high and stroma-low tumors. We observed no significant difference in MIRACLE scores between the stroma-high and stroma-low tumors (median 0.85 vs. 0.85, *p* = 0.263; Figure 4A). In concordance with MIRACLE, using the established TIDE algorithm for predicting immune evasion potential, we failed to demonstrate a difference in ICI therapy responsivity between stroma-high and stroma-low tumors (*p* = 0.257; Figure 4B). MIRACLE and TIDE prediction scores demonstrated a weak negative correlation coefficient (rho = −0.142, *p* = 0.009; Figure 4C).

Considering the nearly identical distribution of TIICs in stroma-high and stroma-low tumors (Figure 3A), we analyzed whether MIRACLE scores were associated with total immune cell infiltration, and found a significant positive correlation (rho = 0.423, *p* < 0.001; Figure 4D). Subsequently, we hypothesized that the combination of the TSR and immune cell infiltrate provides for a superior predictive biomarker. To test our hypothesis, we defined four stromal categories based on stromal content, using the TSR, and total immune cell infiltrate, as computed by CIBERSORTx. Representative histological images for the newly defined stromal categories can be found in Figure 4E. Total immune cell infiltration was not significantly different between the stroma-low/immune-high (SLIH, *n* = 107) group and the stroma-high/immune-high (SHIH, *n* = 93) group (median 2.39 vs. 2.45, *p* = 0.47; Figure 4F). The SLIH and SHIH groups demonstrated higher MIRACLE scores than the stroma-low/immune-low (SLIL, *n* = 107) and stroma-high/immune-low (SHIL, *n* = 61) groups (Figure 4G). In addition, the SLIH tumors showed higher MIRACLE scores than the SHIL tumors (Figure 4G). However, there was no significant difference in MIRACLE scores between the SHIH and SLIH groups (median MIRACLE score 0.88 vs. 0.87, *p* = 0.687), suggesting that both groups predict response to ICI therapy equally well. Likewise, there were no significant differences in total TIICs or MIRACLE scores between the SLIL and SHIL groups. In accord with these observations, gene expression of T-cell exhaustion markers only demonstrated significant differences between the immune-high and immune-low groups (Supplementary Figure S3).

Since MIRACLE was recently postulated to complement the established TIDE algorithm for ICI therapy response prediction, we also analyzed TIDE prediction scores for our TME classification [40,41]. Interestingly, although the TIDE prediction scores were not informative between the immune-high and the immune-low groups, the SHIL group demonstrated a higher TIDE prediction score than the SLIL group (median score 0.013 vs. −0.010, *p* = 0.014; Figure 4H), indicating increased immune evasion potential of the SHIL tumors in comparison to the SLIL tumors. The TIDE prediction scores were not significantly different between the SLIH and SHIH groups.

### 3.5. SLIH Tumors Are Associated with Current Biomarkers for ICI Therapy Response Prediction

**Figure 5:**
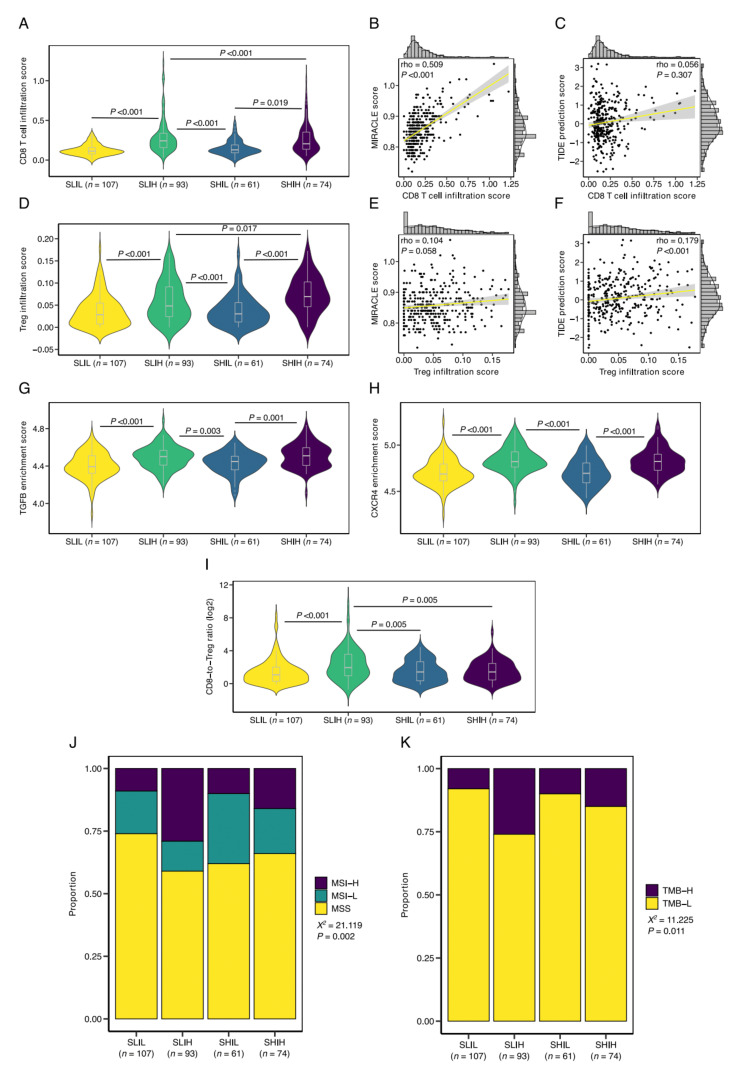
The stromal categories and current biomarkers for ICI therapy response prediction. (**A**) CD8 T cell infiltration in the newly defined stromal categories. Correlation plots of CD8 T cell infiltration and (**B**) MIRACLE scores, and (**C**) TIDE prediction scores. (**D**) Stromal categories and T regulatory cell (Treg) infiltration. Correlation plots of Treg infiltration and (**E**) MIRACLE scores, and (**F**) TIDE prediction scores. Enrichment analysis of (**G**) the TGF-β and (**H**) the CXCR4 signaling pathways. (**I**) Stromal categories and the CD8-to-Treg ratio. (**J**) Proportion of the microsatellite subgroups, stratified by stromal categories. (**K**) Proportion of the TMB subgroups, stratified by stromal categories. Rho, Spearman’s rho; SLIL, stroma-low/immune-low; SLIH, stroma-low/immune-high; SHIL, stroma-high/immune-low; SHIH, stroma-high/immune-high; MSS, microsatellite stable; MSI-L, microsatellite instability-low; MSI-H, microsatellite instability-high; *X*^2^, chi-squared test; TMB-H, tumor mutational burden-high; TMB-L, tumor mutational burden-low.

Next, in light of our earlier observation of increased Treg infiltration in stroma-high tumors, we assessed quantitative differences in CD8 T cell and Treg infiltration between the newly defined stromal groups. CD8 T cell infiltration was significantly different between SLIH and SHIH groups (median 0.242 vs. 0.205, *p* < 0.001) and the immune-high and the immune-low groups (*p* < 0.001; Figure 5A). CD8 T cell infiltration was positively correlated to MIRACLE score (rho = 0.509, *p* < 0.001; Figure 5B) but not to TIDE prediction score (rho = 0.056, *p* = 0.307; Figure 5C). Interestingly, Treg infiltration was increased in the SHIH versus the SLIH group (0.069 vs. 0.048, *p* = 0.017; Figure 5D), and significantly correlated to the TIDE prediction score (rho = 0.179, *p* < 0.001; Figure 5F), but not to the MIRACLE score (rho = 0.104, *p* = 0.058; Figure 5E).

To further address this difference in Treg infiltration, we performed enrichment analysis of the TGF-β and CXCR4 signaling pathways that were previously demonstrated to promote the differentiation of CD4 T cells into Tregs [47,48]. Although we observed significant enrichment of the TGF-β and CXCR4 signaling pathways in the immune-high versus the immune-low groups, enrichment of the signaling pathways was not significantly different between the SHIH and SLIH groups (Figure 5G,H). This finding suggests that the observed differences in CD8 T cell and Treg infiltration between the SHIH and SLIH groups cannot be readily explained by TGF-ß and CXCR4 signaling and are likely driven by other factors. However, key genes from the TGF-β and CXCR4 signaling pathways are included in the CIBERSORTx LM22 signature, used here to define the stromal categories. Therefore, due to the nature of our hybrid transcriptomic classification, caution is warranted in interpreting the results from gene enrichment analyses. Nevertheless, the difference in CD8 T cell and Treg infiltration consequently resulted in an increased CD8-to-Treg ratio in the SLIH group in comparison to the SHIH group (median ratio 1.95 and 1.42, respectively, *p* = 0.005; Figure 5I), a biomarker that was recently found to be predictive of ICI therapy response in non-small cell lung cancer (NSCLC) [49].

In addition to the CD8-to-Treg ratio, MSI has been proposed as a predictive biomarker for response to ICI therapy. As described above, there was no significant association between MSI and the TSR (Figure 3E). We then tested whether MSI was related to the newly defined stromal categories. Notably, in concordance with our finding of an increased CD8-to-Treg ratio in SLIH tumors, the SLIH tumors demonstrated the largest proportion of MSI-H tumors (*X*^2^ = 21.119, *p* = 0.002; Figure 5J). Lastly, we tested whether the stromal categories were associated with TMB, a genomic parameter closely related to MSI. Mutational load data were available for a subset of the tumors (*n* = 268). Similar to MSI, SLIH tumors demonstrated the largest proportion of TMB-H tumors (*X*^2^ = 11.225, *p* = 0.011; Figure 5K). Interestingly, despite the enrichment of MSI-high and TMB-high tumors in the SLIH category, MSI-H and TMB-H tumors were present across all stromal categories. This suggests that the stromal categories defined here may provide additional predictive information that is not captured by current biomarkers for ICI therapy response prediction.

### 3.6. Validation in an External Cohort

Lastly, we aimed to validate our findings in an external cohort of 106 colon adenocarcinoma samples, previously reported by Vasaikar et al. [30]. Patient and tumor characteristics of the validation cohort can be found in Supplementary Table S2. Due to a lack of datasets containing both whole transcriptome sequencing data and histological tissue slides in colon cancer, we were not able to mimic the analyses performed in the discovery cohort. However, since we recently reported an association between histologic stromal content and the transcriptomic ESTIMATE algorithm for tumor purity, we then decided to simulate the stromal categories using said algorithm [17,50]. To define the four stromal categories, we utilized ESTIMATE’s stromal score to categorize tumors as stroma-high or stroma-low, maintaining the same distribution as in the discovery cohort (40.3% stroma-high, 59.7% stroma-low). In addition, ESTIMATE’s immune score was used to categorize tumors as either immune-high or immune-low.

**Figure 6:**
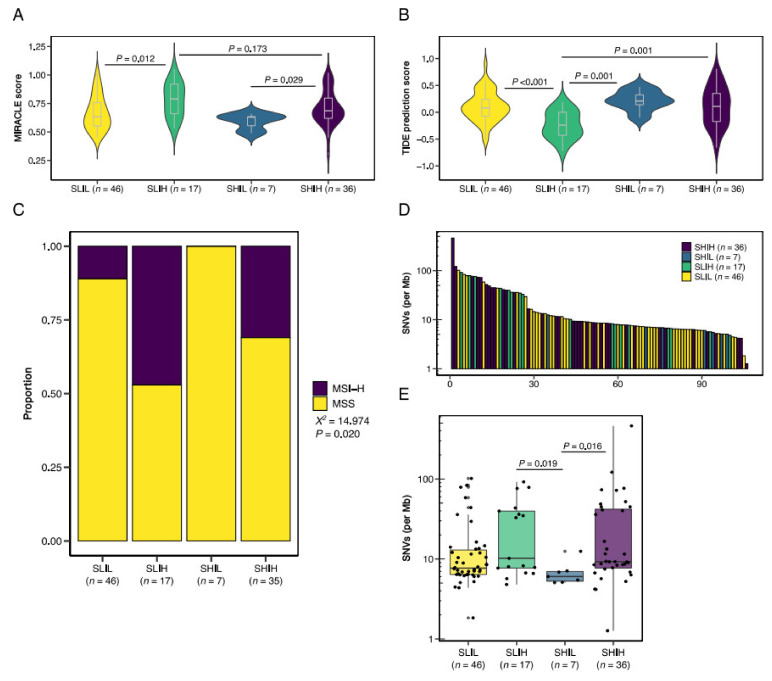
Validation of the stromal categories in an external cohort. The stromal categories and (**A**) MIRACLE scores, and (**B**) TIDE prediction scores. (**C**) Proportions of microsatellite status subgroups per stromal category. (**D**) Bar chart of single nucleotide variant (SNV) rate per sample. (**E**) SNV rate per stromal category. SLIL, stroma-low/immune-low; SLIH, stroma-low/immune-high; SHIL, stroma-high/immune-low; SHIH, stroma-high/immune-high. MSS, microsatellite stable; MSI-H, microsatellite instability-high; *X*^2^, chi-squared test.

We then computed MIRACLE and TIDE prediction scores for the categorized tumors. In concordance with the discovery cohort, the immune-high tumors demonstrated the highest MIRACLE scores but were not significantly different between the SLIH and the SHIH groups (Figure 6A). Interestingly, although not discriminative in the discovery cohort, the TIDE prediction scores in the validation cohort were the lowest in the SLIH group (Figure 6B), suggesting that these tumors demonstrate the least immune evasion potential of the stromal categories. We then noticed a trend of increased TIDE prediction scores in the SHIL tumors in comparison to the SLIL tumors; however, the TIDE prediction scores were not significantly statistically different, possibly due to the small sample size of the SHIL group.

In addition to relatively low TIDE prediction scores, the SLIH stromal category contained the highest proportion of MSI-H tumors (*X*^2^ = 14.974, *p* = 0.020; Figure 6C). We then compared single nucleotide variants (SNV) between the different stromal categories and observed extensive heterogeneity (Figure 6D). Although the SNV rate in both immune-high groups was significantly different from the SHIL group, we did not observe significant differences between the immune-high groups and the SLIL group (Figure 6E). Nevertheless, although based on transcriptomic categorization only, the results in the validation cohort provide further support for the TME classification as a predictive biomarker for immunotherapy response.

## 4. Discussion

In the current work, we aimed to characterize the TIIC composition in stroma-high and stroma-low tumors using bioinformatics approaches. Recent advances in onco-immunology have established TIICs as novel targets in cancer therapeutics, resulting in the widespread application of ICI therapy in solid primary tumors. To account for interpatient variability in treatment response and optimize precision medicine, detailed characterization of the TME is warranted. Recently, the previously proposed TMB biomarker for ICI therapy response was proven to be predictive in only a subset of primary tumor types [26]. In addition, an explorative study of neoadjuvant ICI therapy in early-stage colon cancer demonstrated pathologic responses in both MSS and MSI tumors [51]. The recent emergence of computational oncology has given rise to algorithms that accurately predict ICI therapy response based on pre-treatment tumor profiles, but require thorough transcriptomic analyses that have not been adopted in clinical practice so far [40,41]. These findings highlight the need for a clinically feasible biomarker capable of accurate prediction of response to ICI therapy. Here, we demonstrate how a combination of two quantitative TME parameters, stromal content and total immune cell infiltration, correlate to multiple previously established predictive biomarkers of ICI therapy response, and may provide a stromal alternative to the conventional malignant-cell-oriented biomarkers, such as microsatellite status, TMB, and PD-L1 expression.

The TSR has been recognized as an independent predictor of survival in a multitude of epithelial malignancies and is currently subject to prospective validation for colon cancer in the international UNITED study [34,35]. At the molecular level, histologically defined stroma-high tumors demonstrated increased expression of CAF markers when compared to stroma-low tumors [18]. Moreover, a recent functional report on CAF subtypes described an important role for CAFs in the attraction of Tregs towards the TME, thereby contributing to a local immune-suppressive environment [47,52]. Interestingly, we observed increased infiltration of Tregs in stroma-high tumors, one of few immune-compositional differences between stroma-high and stroma-low tumors. Nevertheless, when we interrogated the TSR to the recently developed MIRACLE score and the previously established TIDE prediction score, we did not observe a discriminative capacity of the TSR in predicting ICI therapy response [40,41]. Apart from its prognostic value in solid primary tumors, it is therefore likely that stromal-content, on its own, is insufficient for predicting ICI therapy response.

The nearly identical distribution of total TIICs in stroma-high and stroma-low tumors observed here led us to define a TME classification based on the combined assessment of stromal content and immune cell infiltration. MIRACLE scores were higher in the immune-high tumors in comparison to the immune-low tumors. The MIRACLE score did not, however, discriminate between the SLIH and SHIH groups, suggesting that these groups show comparable responsivity to ICI therapy. However, when we evaluated the complementary TIDE prediction score, an algorithm that captures the immune evasion potential of the tumor, we noticed a decreased responsivity to ICI therapy in the stroma-high subgroups versus the stroma-low subgroups in both the discovery and validation cohort. This suggests that stromal content affects ICI therapy responsivity, and that combined assessment of stromal content and immune infiltration may provide for a superior biomarker compared to either singular parameter. Of note, a particularly interesting observation in the discovery cohort was an increased TIDE prediction score, surrogate for increased immune evasion potential, in the SHIL tumors versus the SLIL tumors. This suggests that stromal content is likely to be involved in distinct mechanisms of immune evasion. Insight into these distinct immune evasion mechanisms may increase our understanding of the determinants of tumor immunogenicity and should be the subject of future studies.

The results described here do not stand on their own. Classification of TME subtypes has been proposed as a capable predictor of ICI therapy response [38,53]. Recently, a comprehensive analysis of gene expression profiles from >10,000 tumor samples across 20 primary tumor types identified four conserved pan-cancer TME subclasses based on previously published stromal and immune gene signatures [53]. The TME subclasses were found to be associated with response to immunotherapy. Notably, the immune-enriched, non-fibrotic TME subclass, an equivalent to our SLIH stromal group, demonstrated the highest responsivity to immunotherapy, a finding that supports the results described here. In addition, the SLIH tumors in this study were associated with current biomarkers for ICI therapy response prediction, namely MSI, TMB, and the CD8-to-Treg infiltration ratio. We therefore postulate that SLIH tumors exhibit the highest responsivity to ICI therapy and are likely to demonstrate the highest response rates of the four stromal categories.

In contrast to the transcriptomic classification by Bagaev et al., we defined a hybrid TME classification of histologically quantified stromal content and transcriptome deconvoluted immune cell infiltration [53]. Ultimately, for feasible clinical application, we aim to validate our findings and develop a standardized approach to stromal content and immune cell infiltration, using histological quantification only. Recent reports by our group found significant correlations between stromal gene expression and histological stromal content [17,50]. Indeed, here we found comparable results between the histological-defined discovery cohort and the transcriptomic-defined validation cohort. However, it remains to be tested how transcriptomic-derived immune cell infiltration relates to standardized histological TIIC quantification, which should be the subject of future studies.

There are some limitations to our study. The TME classification described here comprises a hybrid classification based on histologic and transcriptomic quantification data. For clinical application, an inexpensive, easily applicable, and time-efficient tool, such as a singular histological approach similar to the current TSR, is desirable. In addition, the categorization into immune-high and immune-low groups was arbitrarily based on a median cut-off of the total TIIC infiltration. For improved performance, an optimal cut-off value should be investigated in future studies. Of note, pathway analyses had limited interpretability due to overlap between immune-related gene sets and the CIBERSORTx LM22 signature used to categorize tumors in this study. Therefore, we did not perform comprehensive immune pathway analyses. Lastly, although a similar TME classification was recently validated in a pan-cancer cohort, due to the explorative nature of this study, the TME classification requires further validation in ICI-therapy-treated colon cancer cohorts [53].

The current work provides a compact overview of immune cell infiltration in histological stroma-high and stroma-low tumors. We report an increased infiltration of Tregs in stroma-high tumors, and a lack of discriminative capacity of the TSR as a single parameter in predicting the response to ICI therapy. Consequently, a newly defined TME classification based on the combined assessment of stromal content and immune cell infiltration was associated with previously established biomarkers and improved the likelihood of predicting ICI therapy response. We postulate that stroma-low/immune-high tumors demonstrate the highest responsivity to ICI therapy. Although further validation is warranted, a combined assessment of the TSR and tumor immune cell infiltration could potentially serve as an easy-to-use predictor of ICI therapy response and guide the treatment decision-making process accordingly. In addition, the biomarker described here could provide a stromal alternative to conventional malignant-cell-oriented biomarkers for ICI therapy response prediction, such as microsatellite status, TMB, and PD-L1 expression. Future studies should focus on the clinical significance of the TME classification in immunotherapy-treated patient cohorts.

## Data Availability

RNA-seq data used in this study is publicly available on The Cancer Genome Atlas (TCGA) COAD project (https://portal.gdc.cancer.gov/, accessed on 1 June 2021). Patient/sample identifiers used in this study are provided in the supplementary data. All novel computed scores in this study are provided in the supplementary data. Any additional data are available from the corresponding author upon reasonable request.

## Supplementary Materials

The following are available online at https://www.mdpi.com/article/10.3390/cells10112935/s1, Figure S1: Flowchart of tumor–stroma ratio (TSR) scoring, Figure S2: Checkpoint expression and the TSR, Figure S3: Checkpoint expression and the stromal categories, Table S1: Immune cell phenotypes included in the CIBERSORTx LM22 signature, Table S2: Baseline patient and tumor characteristics for the discovery and validation cohorts, Table S3: Baseline characteristics of the stroma-low and stroma-high populations, as scored by the TSR, Data S1: TCGA COAD patient identifiers and novel computed scores.
