# Supplementary material for: Combined Assessment of the Tumor–Stroma Ratio and Tumor Immune Cell Infiltrate for Immune Checkpoint Inhibitor Therapy Response Prediction in Colon Cancer
Source: Cells. 2021 Oct 28;10(11):2935. doi: 10.3390/cells10112935 (PMC8616493; doi:10.3390/cells10112935)
Supplement: Supplementary file 1 [file cells-10-02935-s001.zip › Supplementary_figures_tables.pdf]

Supplementary Materials

Combined Assessment of the Tumor–Stroma Ratio and Tumor Immune Cell Infiltrate for Immune Checkpoint Inhibitor Therapy Response Prediction in Colon Cancer

Cor J. Ravensbergen 1, Meaghan Polack 1, Jessica Roelands 2, Stijn Crobach 2, Hein Putter 3, Hans Gelderblom 4, Rob A. E. M. Tollenaar 1,† and Wilma E. Mesker 1,*,†

1 Department of Surgery, Leiden University Medical Center, Albinusdreef 2, 2300RC Leiden, The Netherlands; c.j.ravensbergen@lumc.nl (C.J.R.); m.polack@lumc.nl (M.P.); R.A.E.M.Tollenaar@lumc.nl (R.A.E.M.T.)
2 Department of Pathology, Leiden University Medical Center, Albinusdreef 2, 2300RC Leiden, The Netherlands; j.p.roelands@lumc.nl (J.R.); a.s.l.p.crobach@lumc.nl (S.C.)
3 Department of Medical Statistics, Leiden University Medical Center, Albinusdreef 2, 2300RC Leiden,
The Netherlands; h.putter@lumc.nl
4 Department of Medical Oncology, Leiden University Medical Center, Albinusdreef 2, 2300RC Leiden,
The Netherlands; A.J.Gelderblom@lumc.nl
* Correspondence: w.e.mesker@lumc.nl; Tel: +31-715262987
† Authors equally contributed to this work.

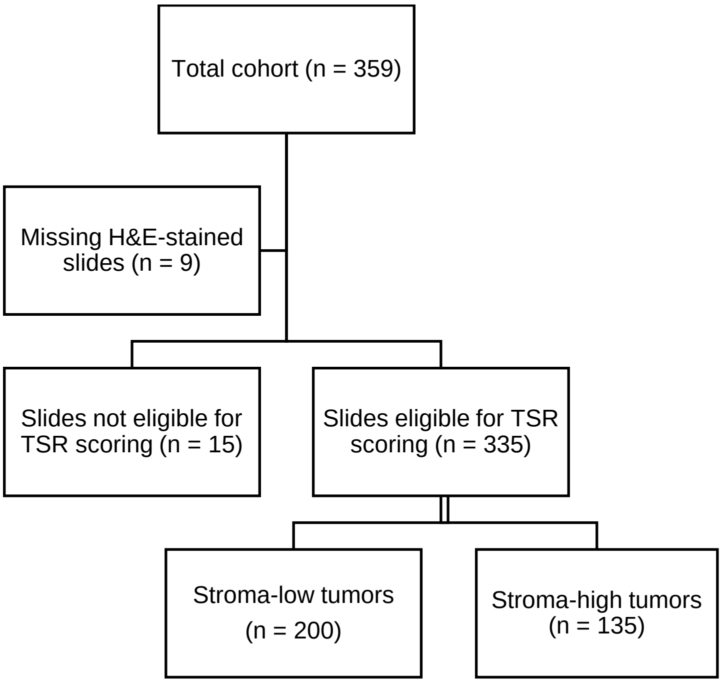

Supplementary Figure S1 [online only]. Flowchart of tumor-stroma ratio (TSR) scoring of the TCGA COAD cohort. *H&E, hematoxylin & eosin.*

Supplementary Table S1 [online only]. Immune cell phenotypes that can be inferred by the CIBERSORTx LM22 547-gene signature matrix.

CIBERSORTx LM22 signature
B cells memory
B cells naive
Dendritic cells activated
Dendritic cells resting
Eosinophils
Macrophages M0
Macrophages M1
Macrophages M2
Mast cells activated
Mast cells resting
Monocytes
Neutrophils
NK cells activated
NK cells resting
Plasma cells
T cells CD4 memory activated
T cells CD4 memory resting
T cells CD4 naive
T cells CD8
T cells follicular helper
T cells gamma delta
T cells regulatory

Supplementary Table S2 [online only]. Baseline patient and tumor characteristics for the TCGA COAD discovery and CPTAC validation cohorts.

	**TCGA COAD (*n* = 359)**	**CPTAC (*n* = 106)**
Age (%)		
<60	97 (27.0)	35 (33.0)
60-75	156 (43.5)	48 (45.3)
>75	106 (29.5)	21 (19.8)
Missing	0 (0)	2 (1.89)
Gender (%)		
Female	169 (47.1)	63 (59.4)
Male	190 (52.9)	43 (40.6)
Histologic subtype (%)		
Adenocarcinoma	359 (100)	106 (100)
Other	0 (0)	0 (0)
pTNM (%)		
I	62 (17.3)	12 (11.3)
II	147 (40.9)	42 (39.6)
III	98 (27.3)	45 (42.5)
IV	52 (14.5)	7 (6.60)
Neoadjuvant therapy (%)		
Yes	0 (0)	0 (0)
No	359 (100)	106 (100)
Microsatellite status		
MSS	236 (65.7)	81 (76.4)
MSI-Low	66 (18.4)	0 (0)
MSI-High	57 (15.9)	24 (22.6)
Missing	0 (0)	1 (0.94)

Supplementary Table S3 [online only]. Baseline characteristics of the stroma-low and stroma-high populations from the TCGA COAD cohort, as scored by the tumor-stroma ratio (TSR).

	**Stroma-low (*n* = 200)**	**Stroma-high (*n* = 135)**	***P*-value**
Age (%)			
<60	47 (23.5)	44 (32.6)	0.420†
60-75	93 (46.5)	54 (40.0)	
>75	60 (30.0)	37 (27.4)	
Gender (%)			
Female	94 (47.0)	73 (54.1)	0.231‡
Male	106 (53.0)	62 (45.9)	
pTNM (%)			
I	37 (18.5)	22 (16.3)	0.652†
II	79 (39.5)	55 (40.7)	
III	55 (27.5)	39 (28.9)	
IV	29 (14.5)	19 (14.1)	
Microsatellite status (%)			
MSS	134 (67.0)	87 (64.4)	0.967†
MSI-Low	29 (14.5)	30 (22.2)	
MSI-High	37 (18.5)	18 (13.3)	

†Chi-squared test

‡Fisher's exact test
